# Performance of a Heating Block System Designed for Studying the Heat Resistance of Bacteria in Foods

**DOI:** 10.1038/srep30758

**Published:** 2016-07-28

**Authors:** Xiao-xi Kou, Rui Li, Li-xia Hou, Zhi Huang, Bo Ling, Shao-jin Wang

**Affiliations:** 1 College of Mechanical and Electronic Engineering, Northwest A&F University, Yangling, Shaanxi 712100, China; 2 Department of Biological Systems Engineering, Washington State University, Pullman, WA 99164-6120, USA

## Abstract

Knowledge of bacteria’s heat resistance is essential for developing effective thermal treatments. Choosing an appropriate test method is important to accurately determine bacteria’s heat resistances. Although being a major factor to influence the thermo-tolerance of bacteria, the heating rate in samples cannot be controlled in water or oil bath methods due to main dependence on sample’s thermal properties. A heating block system (HBS) was designed to regulate the heating rates in liquid, semi-solid and solid foods using a temperature controller. Distilled water, apple juice, mashed potato, almond powder and beef were selected to evaluate the HBS’s performance by experiment and computer simulation. The results showed that the heating rates of 1, 5 and 10 °C/min with final set-point temperatures and holding times could be easily and precisely achieved in five selected food materials. A good agreement in sample central temperature profiles was obtained under various heating rates between experiment and simulation. The experimental and simulated results showed that the HBS could provide a sufficiently uniform heating environment in food samples. The effect of heating rate on bacterial thermal resistance was evaluated with the HBS. The system may hold potential applications for rapid and accurate assessments of bacteria’s thermo-tolerances.

Knowledge of bacteria’s heat resistance is of great importance in developing effective food processing methods. Selection of appropriate heating parameters can effectively reduce the target microorganism populations while achieving minimal thermal impacts on food quality. In addition to growth temperatures, stages of growth, bacterial strains, sample compositions, and the pH of the heating medium, heat resistance of bacteria is mainly affected by final temperature, holding time and heating rate^1^,^2^,^3^. Thermal death time (TDT) test is a common method to determine the heat resistance of bacteria^1^,^4^,^5^,^6^,^7^. The device used for those TDT tests needs to meet the basic requirements, such as precisely controlled sample temperatures, a short come-up time (CUT) to avoid thermal adaptation of bacteria, and isothermal conditions for uniform sample temperature distributions^5^. Therefore, choosing an appropriate test method is important to obtain accurate determination of bacteria’s heat resistance.

There have been many studies on TDT test devices in foods. Odlaug *et al.*^8^ used aluminum tubes in a miniature retort system to study thermal destruction of *Clostridium botulinum* spores in tomato juice. Gaze *et al.*^9^ placed samples of ground meat in 0.5- to 1-mm thick sterile bags, and then immerged them in a shaking water bath. Kotrola *et al.*^10^ placed turkey meat samples into a TDT sealed tube heated by a shaking oil bath. Chung *et al.*^11^ designed an aluminum test cell heated in oil bath for determining the heat resistance of *Clostridium sporogenes* in selected foods. There were also studies with capillary tubes using water bath for apple juice^12^, and glass vials with an oven-heated sand bath for crushed cocoa bean and hazelnut shells to determine the bacteria’s thermo-tolerance^13^. Although these devices are common tools with water or oil bath for characterizing heat-resistant bacterial spores in foods, various thermo-tolerances of the same bacatria have been frequently reported from different heating methods^4^,^6^,^14^,^15^,^16^. Foster *et al.*^17^,^18^ developed a new apparatus to control sample surface temperatures during rapid heating and cooling cycles. This system used hot air and steam as the heating medium to provide a fast “dry” or “wet” heat treatment to a single food sample by setting a starting temperature, end temperature, heating time, hold time and cooling time by a user-friendly software^19^,^20^. This system could be improved by using the sample core temperature to cover the bacteria’s resistance over the whole volume and multiple samples to accelerate the experimental process.

Heating rates have been shown to have a significant effect on thermo-tolerance of bacteria. Slow heating rates often result in enhanced heat resistance of bacteria with large *D*-values at the same target temperatures^11^,^21^,^22^,^23^,^24^. These differences in bacteria’s heat resistance are most likely the result of acclimation and physiological adjustment during the slower heating, most probably caused by the production of heat shock protein^25^,^26^. However, heating rates in the above-mentioned devices are only dependent on thermal properties of devices and food materials, and could not be controlled during the experiment. The different heat resistance of *Escherichia coli* has been reported by Khoo *et al.*^27^ and Büchner *et al.*^5^ due to using different device materials, such as glass and aluminum. The *D*-values of *Salmonella* at 60 °C are clearly different in 1-g and 3-g samples^28^,^29^. Various heat resistances of the same bacteria are also reported in different food materials^30^,^31^,^32^. The common thermal treatments, such as hot air^33^, water^22^,^34^, steam^35^, radio frequency^36^,^37^,^38^, microwave^39^,^40^, infrared^41^, and ohmic heating^42^, have been widely used for pasteurization and sterilization in foods. These thermal treatments would not meet the required efficacy if developing based on the thermal death kinetic data of target bacteria under a fixed heating rate obtained from the above-mentioned devices. Therefore, designing a heating block system (HBS) to simulate the heating rates is needed in developing an effective thermal treatment to control bacteria in food using hot air/water, microwave, and radio frequency energy.

The temperature-time history in a designed HBS could be easily measured by thin thermocouples but the heating uniformity is difficult to evaluate in a small volume of the TDT cell by measurement. Computer simulation and mathematical modeling may serve as a valuable tool for rapid analysis of heating uniformity in foods. For example, Chung *et al.*^11^ used FEMLAB to evaluate the performance of aluminum test cells designed for determining the heat resistance of bacterial spores in foods. Yan *et al.*^43^ applied a validated computer model using COMSOL to evaluate the heating performance of the HBS for studying insect thermal death kinetics and optimizing thermal treatment conditions when modified the HBS to include controlled atmospheres. Ben-Lalli *et al.*^44^ defined 2D axial-symmetric domains during both convective and microwave heating treatments, and obtained 95% accuracy between the simulated temperatures and experimental data. Huang *et al.*^45^,^46^ developed a 3-D theoretical model using COMSOL to determine differential heating of insects in soybeans when subjected to radio frequency treatments. Therefore, the finite element computer simulation may provide a useful tool to analyze the heating transfer in the designed HBS and validate the performance and uniformity of the HBS.

Objectives of this study were to: (1) design a TDT HBS suitable for studying heat resistance of bacteria, (2) evaluate the performance of the HBS with five different food samples under three heating rates, (3) analyze the heating uniformity in a designed HBS by the validated simulation model using finite element software COMSOL, and (4) apply this system to investigate the thermal inactivation of *Escherichia coli* at 57 °C under five heating rates.

## Methods

### The TDT HBS design

**Figure 1:**
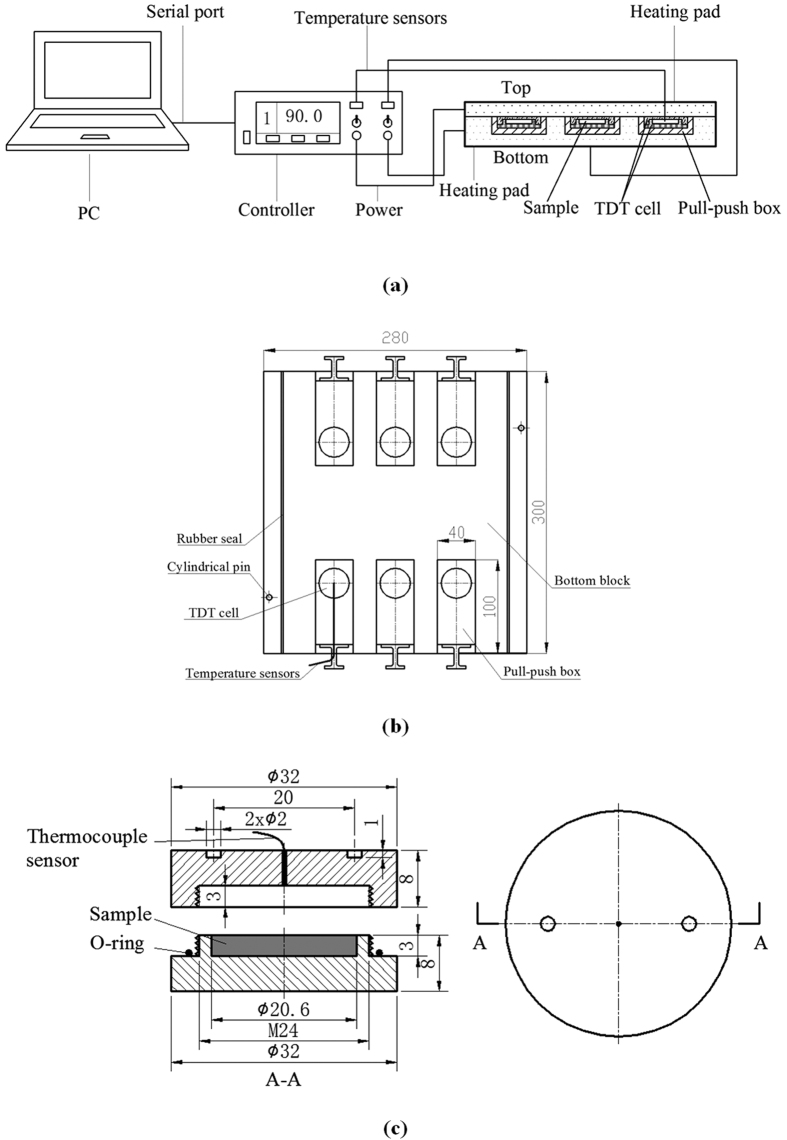
Schematic diagram of (**a**) the TDT HBS, (**b**) top view after removing the top block and (**c**) schematic diagram of a TDT test cell with all dimensions in mm.

The HBS consisted of a heating unit, a data acquisition/control unit, and a computer (Fig. 1a). The heating unit included top (28 cm × 28 cm × 1.6 cm) and bottom (28 cm × 30 cm × 2.4 cm) blocks, heating pads, and 6 pull-push boxes with 6 TDT cells as previously used^11^,^21^. The heating blocks were made of aluminum alloys with low heat capacitance (903 J/kg°C) and high thermal conductivity (234 W/m°C) to provide smooth block temperatures over the heating and holding periods^11^,^47^. Eight custom-made heating pads (250 W) were glued on the top and bottom block surfaces, providing a maximum heating flux density of 12000 W/m^2^. Calibrated type-T thermocouples (TMQSS-020-6, Omega Engineering Ltd., CT, USA) inserted through sensor paths were used to monitor temperatures of the top and bottom blocks, and one sample temperature in a TDT cell. Heating rate (0.1 to 13.3 °C/min) and the set-point temperature (maximum of 120 °C) were controlled by the Visual Basic software via a solid-state relay. Two proportional-integral-derivative (PID) controllers (I32, Omega Engineering, Inc., Stamford, CT, USA) regulated the two block surface temperatures separately.

The 6 TDT cells were just fitted in the pull-push boxes, which were located in the bottom block and distributed uniformly (Fig. 1b). The box could be easily pushed into the block for heating and pulled out for fast cooling in ice water. The TDT cell consisted of a base and a screwed-on cap to allow easy loading and unloading of the sample (Fig. 1c). A rubber o-ring between the two parts provided a hermetic seal to maintain constant sample moisture content. The disk-shaped cavity was 20.6 mm in diameter and 3 mm high, providing a sample space of 1.00 ml. The design of the TDT cell having a high ratio of heated surface area to sample volume may provide a short CUT^11^.

### Performance evaluation of the HBS

Distilled water, apple juice, mashed potato, almond powder and ground beef were selected as representative liquid, semi-solid and solid foods. During the sample preparation, dry mashed potato flakes (Simplot, Australia) were mixed with distilled water to formulate into 15.38% wet basis (w.b.) mashed potato^11^,^48^. Almond kernels were obtained from the Almond Board of California, Modesto, CA, USA^49^, and kept under refrigeration until use. Kernels were milled with a grinder, and passed through a no. 18 mesh (16 Tyler)^16^. The moisture content of 6% wet basis (w.b.) was determined using the vacuum oven method according to the Association of Official Analytical Chemists standard [method 27.005]. Raw ground beef (90% lean), obtained from alocal store, was placed in zip-lock bags, vacuum-sealed, and frozen at −20 °C^28^.

One ml of distilled water, 1-ml apple juice (Huiyuan 100% Apple Juice, Yangling, China) or 1-g mashed potato, or 0.8-g almond powder, or 1.0-g thawed beef was placed into the TDT cell at room temperature. For water, apple juice and mashed potato, the block temperature was then raised to 90 °C at 1, 5, and 10 °C/min, and held at that temperature for 1 min, enough to kill most bacteria^34^,^50^,^51^,^52^. For almond powder and beef, because of their stability states, the temperature was set to 120 °C, and the heat treatments followed the same procedure. The sample temperature in the TDT cell and the block temperatures were monitored and recorded by the data acquisition/control unit and the computer program. Each treatment was replicated three times.

To further evaluate the performance of the HBS, 1-ml distilled water in the same TDT cell with water bath was used as a reference method for comparisons. The prepared TDT cell was heated up to 70 °C based on the heat resistance of *Escherichia coli*^21^,^53^ in a water bath (SC-15, Ningbo Scientz Biotechnology Co., Ltd., China), maintained at that temperature for 2.4 min, and then cooled immediately in the ice water. The core temperature of the sample was measured using a thin pre-calibrated Type-T thermocouple (TMQSS-020-6, Omega Engineering Ltd., CT, USA) and recorded with a data logger (CR-1000, Campbell Scientific. Inc, Logan, Utah, USA) at a time interval of 6 s, the same as used in the HBS. For the HBS method, the same target temperature of 70 °C with the fastest heating rate of 13.3 °C/min was used to treat the same sample, together with the same procedure for the post-heat treatments as in the water bath method. The experiment was replicated three times.

### Finite element model and simulation

A heat transfer model was mainly used to simulate the heating process of the HBS^43^. The HBS was modeled as a three-component system consisting of two aluminum blocks and six cell samples. The heating pads were set as boundary heat sources. The heat fluxes transferred from heating pads to top and bottom blocks and then to the cell and sample through heat conduction. The heat loss from the side walls of the heating blocks to the environment was estimated by natural convective heat loss with *h* value of 5 W/m^2^ °C^43^. It was assumed in the model that thermal properties of the aluminum block, air and sample, and heat transfer resistances betweenthe blocks or between heating pads and blocks were constant over the test temperature range, which are listed in Table 1.

Transient heat transfer through the block and sample was governed by the following differential equation:


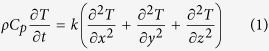


where *ρ* is the mass density of the material in kg/m^3^, *C*_*p*_ is the specific heat capacity in J/kg°C, *k* is the thermal conductivity in W/m°C, *T* is the temperature in °C, *t* is the time in s, and *x*, *y*, or *z* is the Cartesian coordinate position in m. Equation (1) was subjected to the following initial condition:


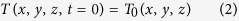


where *T*_*0*_ is the initial temperature of the materials (°C). Heat flux *q* (W/m^2^), from the heating pad, in the direction normal to the interfaces between the top and bottom blocks and the heating pads is described by the following^43^:


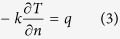


where *n* is the outward normal direction to surface. Convective heat transfer at the block edge normal to the side was given by:


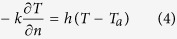


where *h* is the surface heat transfer coefficient in W/m^2^ °C, and *T*_*a*_is the ambient air temperature in °C. The boundary heat source from the heating pads provides different heating flux (*q*_*i*_), which is influenced by heating rates and block thicknesses, and could be calculated as follows:


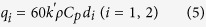


where *k*′ is the heating rates (°C/min), and *d*_*1*_ and *d*_*2*_are the thickness of the top (16 mm) and bottom block (24 mm), respectively.

The temperature uniformity index (TUI) is a useful parameter to evaluate the heating uniformity of treated samples in simulations^45^,^54^:


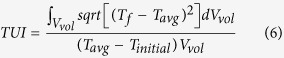


where *T*_*f*_ is the final local temperature in °C, *T*_*avg*_ is the average temperature in °C over the volume (*V*_*vol*_, m^3^), and *T*_*initial*_ is the initial temperature in °C before heating.

A finite element method was used to numerically solve the energy, momentum and transport equations. Physical model was built using Creo software (Creo Parametric 2.0, Parametric Technology Co., Needham, MA, USA). COMSOL Multiphysics software (V4.4a COMSOL Multiphysics, COMSOL, Co., LTD., Shanghai, China) was used to numerically solve the heat transferequations (Equations 1, 2, 3, 4, 5, 6). A relatively fine tetrahedral mesh was created. The mesh size was considered suitable when the temperature difference at the same point between two sequential sets of meshes was less than 1%. The final mesh system consisting of 23,342 domain elements (tetrahedral), 7,220 boundary elements (triangular), 1,404 edge elements (linear), and 210 endpoint elements was adopted in subsequent calculations. The time-dependent solver was used. The time step was set as 0.1 min, and a relative tolerance was 0.01. A LenovoA4600k computer with two Dual Core i5–2400, 3.00 GHz Xeon processors and 4 GB RAM equipping a Windows 8 64-bit operating system was used to run the software. Total solution time varied from 5 to 10 min, depending on the simulation sequence and specific conditions.

### A case study with *Escherichia coli* in mashed potato

*Escherichia coli* ATCC 25922 was selected because it is nonpathogenic and has demonstrated log-linear inactivation kinetics under isothermal conditions. Mashed potato was chosen as a model semisolid food to eliminate heat convection and facilitate placement into TDT cells^11^,^21^.

The *E. coli* strains were obtained from the College of Food Science and Engineering, Northwest A&F University (Yangling, China). 0.8-g mashed potato and 20-μl of *E. coli* bacterial suspensions with 10^9^ CFU/ml cell populations was placed inside each TDT cell. A treatment temperature of 57 °C^21^, and heating rates of 0.1, 0.5, 1, 5, and 10 °C/min were used. When achieving the target temperature, test cells were held for different time intervals, depending on the heating rates, to achieve at least a 5-log reduction. After holding, the test cells were immediately placed in an ice-water bath until further analysis was performed. All the experiments were replicated two times.

### Statistical Analysis

All statistical analyses were performed at a 5% significance level using the Microsoft Excel variance procedure (Microsoft Office Excel2007).

## Results

### Heating performance of the HBS

**Figure 2:**
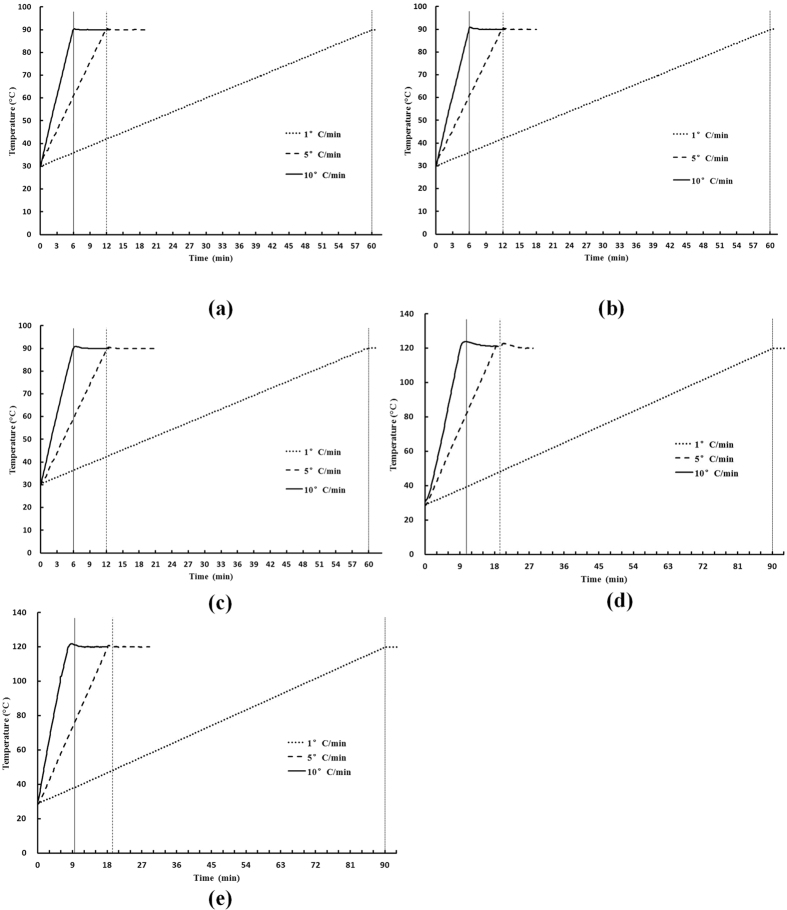
Experimental temperature-time histories of (**a**) water, (**b**) apple juice, (**c**) mashed potato, (**d**) almonds powder and (**e**) beef in the TDT HBS under three heating rates.

Figure 2 shows experimental temperature-time histories of water, apple juice, mashed potato, almond powder and beef in the HBS under three heating rates. The heating rate and final temperature of the HBS were controlled well as reported by Ikediala *et al.*^47^ and Wang *et al.*^23^. Especially in low heating rates and water (juice), the sample temperature in the TDT cell was controlled as expected with small errors and stable final temperatures during holding time due to low thermal inertia. But in mashed potato,almond powder and beef the final sample temperatures had an overshoot of about 1.9, 2.2 and 1.2 °C, respectively, when just achieving the set-point temperature at 10 °C/min. This overshoot was reduced to 0.5 °C within 20 s, which would have little effect on bacteria’s thermal resistance^55^. At the final temperature of the exposure period, food, top and bottom block temperatures deviated from the set point by 0.2, 0.1 and 0.1 °C, respectively.

### Comparison between water bath method and HBS

**Figure 3:**
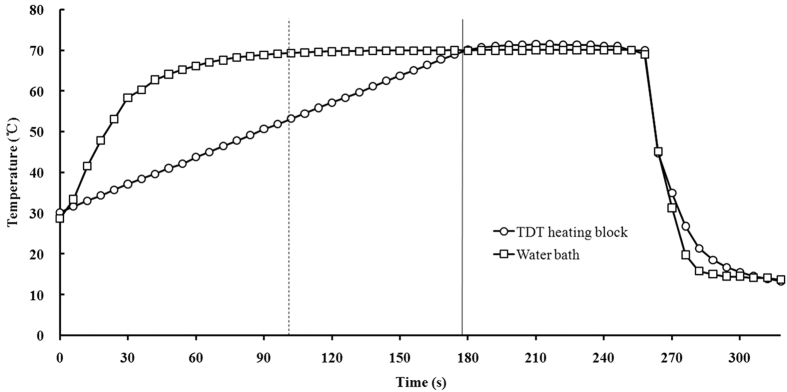
Temperature-time histories of water sample in TDT cells indicating come-up, hold, and cool-down to 10 °C periods both in the TDT HBS and water bath.

Figure 3 shows the comparison of heating processes between the HBS and water bath method. It took 180 s from 30 °C to 70 °C by the HBS in maximum heating rate (13.3 °C/min), with water sample in the TDT cell. Under the same test conditions, the CUT was 108 s for water bath method^16^. The cooling time was similar for both methods, which was in agreement with that in previous studies^11^. The cooling time could be controlled and shortened when using a new refrigeration cooling method as suggested by Foster *et al.*^17^,^18^. Although the HBS’s CUT time was larger than that in the water bath method, the HBS kept the constant heating rates and made it possible to determine the influence of heating rates on bacteria’s heat resistance^56^. This heating rate range in HBS could be enough to simulate the practical thermal treatments for large volume foods. As shown in the temperature-time history from the water bath (Fig. 3), the heating time was about 40 s for the sample’s temperature to increase from 30 °C to 60 °C, but 68 s for rising from 60 °C to 70 °C. The CUT time may change when using the different water bath equipment and food materials. The major advantage of the HBS was that the heating rate in different food could be controlled to simulate the real heating in bulk agricultural products using hot air, hot water and radio frequency energy.

### Finite element simulation and validation

**Figure 4:**
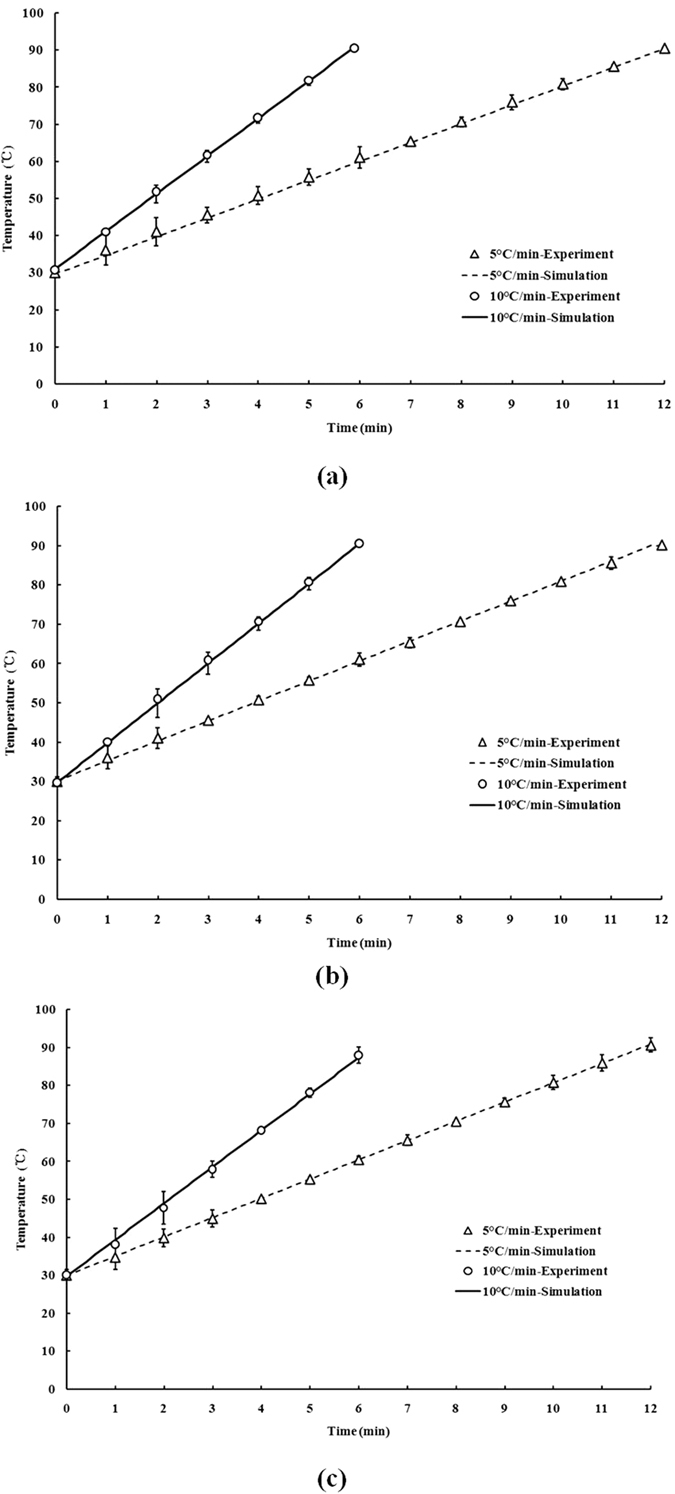
Comparison of temperature–time histories at the center of (**a**) water, (**b**) mashed potato, and (**c**) almonds powder in a TDT cell with the HBS between experiment and simulation.

Figure 4 compares simulated and measured temperature profiles with heating rates of 5 °C/min and 10 °C/min in water, mashed potato, and almond powder. The sample temperature increased lineally with the heating time using the HBS (*R*^*2*^ = 0.99). For all heating rates, simulated data agreed well with experimental temperatures since the maximum and average temperature differences was 0.84 °C and 0.15 °C for water, 0.85 °C and 0.21 °C for mashed potato, 0.88 °C and 0.51 °C for almond powder, respectively, between experiment and simulation during CUT. This illustrates that the boundary conditions and model parameters were adequately setup for estimating actual heat transfers within the HBS, and the validated model could be further used to simulate temperature distributions during the heating process.

**Figure 5:**
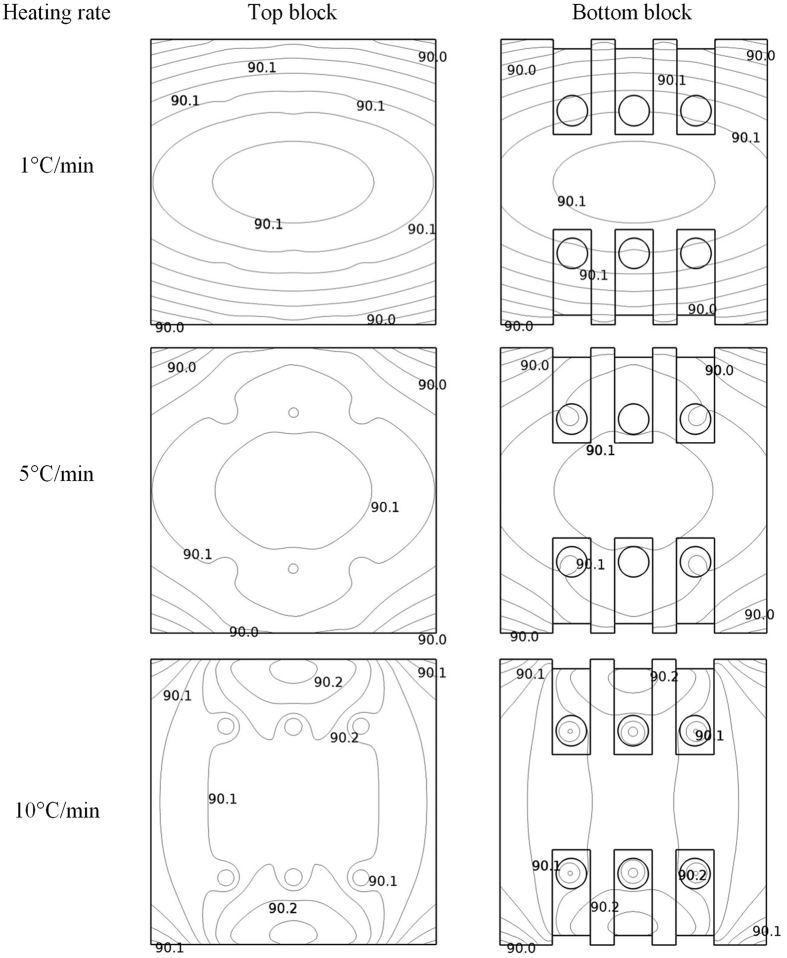
Simulated temperature distributions during the experiment at heating rates of 1, 5 and 10 °C/min for the top and bottom blocks with water samples when the target temperature reached the set-point of 90 °C.

Figure 5 shows the simulated water and block temperature distributions at heating rates of 1 °C/min, 5 °C/min and 10 °C/min when the block temperature just reached the set-point of 90 °C. The simulated surface temperature revealed that the center temperaturewas higher than that of corners and edges due to heat loss at the boundary sidewalls. Especially for the cells’ sections, the surface temperature was the highest and difference from set-point was less than 0.2 °C, suggesting that the thermal process was stable and controlled accurately during the TDT test. Ikediala *et al.*^47^ reported a similar thermal effect with heating blocks. Compared with higher heating rates, the lower heating rates achieved better heating uniformity over the blocks since the TUI decreased by reducing the heating rates from 10 to 1 °C/min. A similar effect of heating rates on the heating uniformity has also been reported by Yan *et al.*^43^. For example, the TUI over the block with the water sample at the set point (90 °C) was 3.55 × 10^−4^, 9.91 × 10^−4^and 1.97 × 10^−3^ with heating rates of 1, 5 and 10 °C/min, respectively. A same trend appeared in the samples, as the TUI in water within the TDT cells was 5.05 × 10^−4^, 2.54 × 10^−3^and 5.14 × 10^−3^ with heating rates of 1, 5 and 10 °C/min (Table 2), respectively.

**Figure 6:**
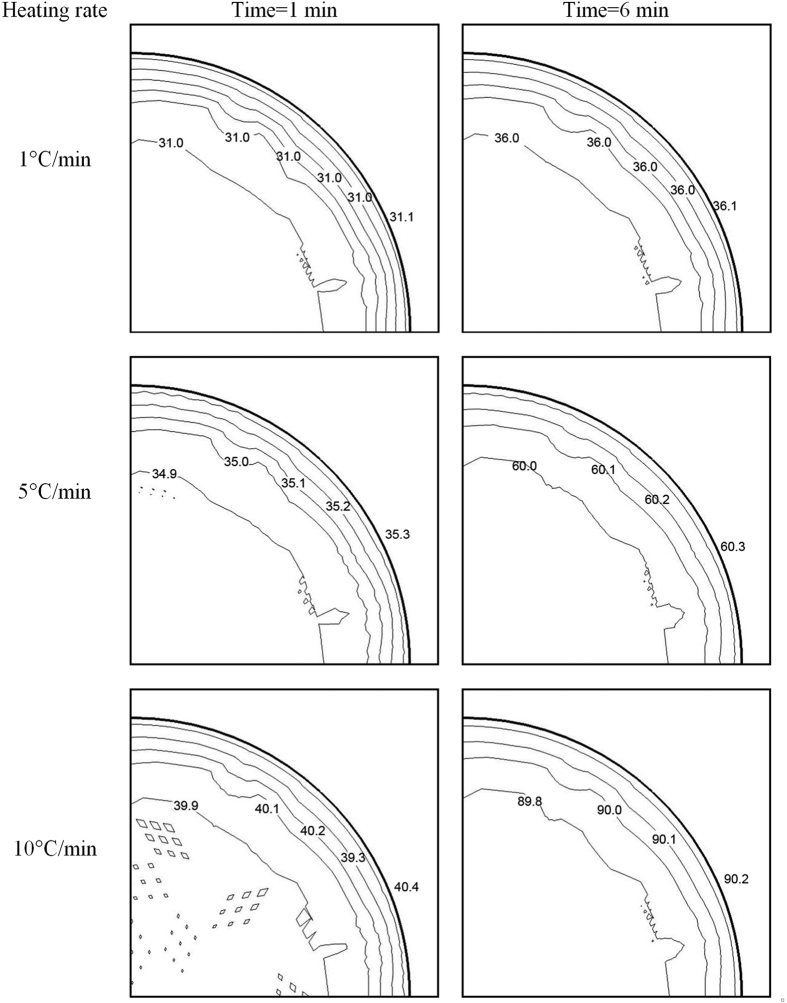
Simulated temperature distributions during the experiment at heating rates of 1, 5 and 10 °C/min for contour plot of a central cross-section of mashed potato samples with heating times of 1 and 6 min from 30 °C.

Figure 6 shows the simulated water temperature contour plot in the TDT cell central cross-section of the HBS under heating rates of 1 °C/min, 5 °C/min and 10 °C/min with heating times of 1 and 6 min, respectively. The results indicated that the outer layer sample temperature was slightly higher (0.1–0.4 °C) than the inner one due to heat conduction from blocks. The heating rate at the center of the test sample estimated from the final temperature and heating time agreed well with the predetermined one. The final sample temperature difference on the cross-section of the TDT cell was less than 0.5 °C. The temperature uniformity trends in TDT cells were similar to those in the blocks as indicated in Fig. 5. But the TUI in the cell was larger than that in blocks due to the similar temperature differences in smaller volume of cells. Compared with higher heating rates, the lower heating rates achieved better heating uniformity in the TDT cells since the TUI decreased by reducing the heating rates from 10 to 1 °C/min (Table 2). For example, the TUI for mashed potato at the set point (90 °C) was 6.03 × 10^−4^, 2.97 × 10^−3^ and 5.99 × 10^−3^ with heating rates of 1, 5 and 10 °C/min, respectively. The higher heat conductivity of the sample achieved the better heating uniformity.

### Effect of heating rate on bacterial thermal resistance with HBS

**Figure 7:**
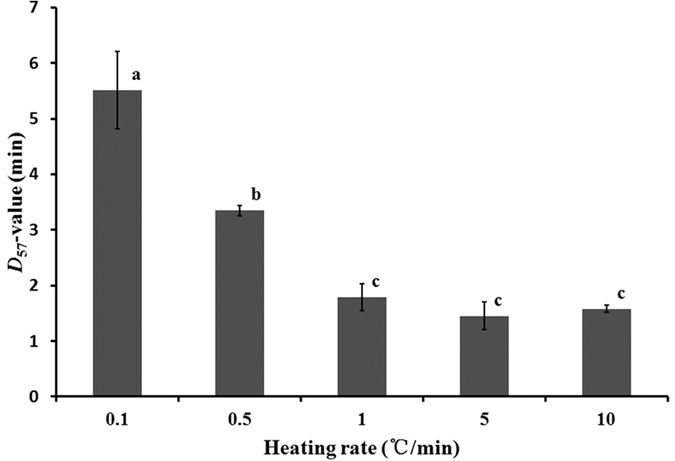
The *D-*values of *E.coli* at 57 °C as influenced by heating rates.

Figure 7 shows the *D*-values of *E.coli* at 57 °C, as influenced by heating rates. The average *D*-value was similar at heating rates of 1,5 and 10 °C/min (P > 0.05), which was in agreement with those (0.96–1.62 min) of the same bacterial in the same material obtained by tube methods with diameters of 3–20 mm^21^. However, the *D*-value at small heating rates (0.1 and 0.5 °C/min) significantly increased from those at higher heating rates (P < 0.05), which was in agreement with observations reported by Chung *et al.*^21^. The thermal resistance of *E.coli* was enhanced at low heating rates for large samples obtained by conventional heating. This may be caused by heat shock proteins produced in bacteria during lengthy exposures to non-lethal temperatures^57^.

## Discussion

The results indicated that the maximum heating rates obtained in HBS were 13.3 °C/min, 13.3 °C/min, 13.3 °C/min, 10.0 °C/min and 11.6 °C/min, respectively, for water, apple juice, mashed potato, almond powder and beef. Although being smaller than those in the apparatus reported by Foster *et al.*^17^,^18^ these heating rates could be enough to simulate thermal resistance of bacteria and thermal behaviors of bulk samples when subjected to hot air, water, and radio frequency heating^22^,^48^,^58^,^59^. The heating rates in foods below the maximum one could be regulated by inputting power of the HBS, which was better than the tube method in water bath with the fixed heating rate^11^,^21^. The finite element computer model could be used for predicting temperature distributions and heating uniformity in various food samples without conducting extensive experiments. Based on the HBS, it can simulate the real sterilization and pasteurization treatments, and is possible to develop a relatively high-temperature-short-time process for thermal sterilization while minimizing quality loss in the host product. The performance of the HBS indicated that this model system could be used for rapid assessment of bacterial thermo-tolerance under different heating rates.

## Conclusions

An unique experimental device, the heating block system (HBS), was developed to heat liquid, semi-solid and solid foods over a wide range of controlled heating rates for studying the inactivation kinetics of bacterial spores. The experimental and simulated results showed that the HBS could provide a sufficiently uniform heating environment in water, apple juice, mashed potato, almonds powder and beef food samples. The good performance of controlling the heating rates, set temperatures, and holding times, may precisely characterize the heat resistance of bacteria in foods to simulate the real sterilization and pasteurization treatments using hot air, hot water and radio frequency energy. The system was used to study thermal resistance of *E. coli* in mashed potato at 57 °C under five different heating rates. The average *D*-value was similar when heating rates above from 1°C/min, but significantly increased at lower heating rates (0.1 and 0.5 °C/min). Further research is needed to confirm the performance of the HBS by determining the effect of heating rates on bacteria’s thermo-tolerance in other foods.

## Additional Information

**How to cite this article**: Kou, X.-x. *et al.* Performance of a Heating Block System Designed for Studying the Heat Resistance of Bacteria in Foods. *Sci. Rep.* **6**, 30758; doi: 10.1038/srep30758 (2016).

## Figures and Tables

**Table 1:** Thermal properties used in the simulation.

Material	Density *ρ* (kg/m^3^)	Specific heat *C*_*p*_ (J/kg°C)	Thermal conductivity *λ* (W/m°C)	Sources
Aluminum block	2702	903	234.00	ref. 47
Water	1000	4180	0.61	ref. 47
Mashed potato (15.4% w.b.)	1050	3460	0.56	ref. 21
Almonds powder (6.0% w.b.)	800	2040	0.18	ref. 60

**Table 2:** Simulated time (min) and heating uniformity (TUI) in three samples within TDT cells using HBS with three heating rates when reaching 90 °C.

	1 °C/min	5 °C/min	10 °C/min
Time (min)			
Water	60.0	12.0	6.0
Mashed potato	60.0	12.0	6.0
Almond powder	60.0	12.0	6.0
TUI (*λ*)			
Water	5.05 × 10^−4^	2.54 × 10^−3^	5.14 × 10^−3^
Mashed potato	6.03 × 10^−4^	2.97 × 10^−3^	5.99 × 10^−3^
Almond powder	1.42 × 10^−3^	7.14 × 10^−3^	1.45 × 10^−2^
